# Cell-free DNA ultra-low-pass whole genome sequencing to distinguish malignant peripheral nerve sheath tumor (MPNST) from its benign precursor lesion: A cross-sectional study

**DOI:** 10.1371/journal.pmed.1003734

**Published:** 2021-08-31

**Authors:** Jeffrey J. Szymanski, R. Taylor Sundby, Paul A. Jones, Divya Srihari, Noah Earland, Peter K. Harris, Wenjia Feng, Faridi Qaium, Haiyan Lei, David Roberts, Michele Landeau, Jamie Bell, Yi Huang, Leah Hoffman, Melissa Spencer, Matthew B. Spraker, Li Ding, Brigitte C. Widemann, Jack F. Shern, Angela C. Hirbe, Aadel A. Chaudhuri

**Affiliations:** 1 Division of Cancer Biology, Department of Radiation Oncology, Washington University School of Medicine, St. Louis, Missouri, United States of America; 2 Pediatric Oncology Branch, Center for Cancer Research, National Cancer Institute, National Institutes of Health, Bethesda, Maryland, United States of America; 3 Division of Biology and Biomedical Sciences, Washington University School of Medicine, St. Louis, Missouri, United States of America; 4 Division of Oncology, Department of Medicine, Washington University School of Medicine, St. Louis, Missouri, United States of America; 5 Siteman Cancer Center, Barnes Jewish Hospital and Washington University School of Medicine, St. Louis, Missouri, United States of America; 6 McDonnel Genome Institute, Washington University in Saint Louis, Missouri, United States of America; 7 Department of Genetics, Washington University School of Medicine, St. Louis, Missouri, United States of America; 8 Department of Pediatrics, Washington University School of Medicine, St. Louis, Missouri, United States of America; 9 Department of Biomedical Engineering, Washington University School of Medicine, St. Louis, Missouri, United States of America; 10 Department of Computer Science and Engineering, Washington University in St. Louis, St. Louis, Missouri, United States of America; University College London, UNITED KINGDOM

## Abstract

**Background:**

The leading cause of mortality for patients with the neurofibromatosis type 1 (NF1) cancer predisposition syndrome is the development of malignant peripheral nerve sheath tumor (MPNST), an aggressive soft tissue sarcoma. In the setting of NF1, this cancer type frequently arises from within its common and benign precursor, plexiform neurofibroma (PN). Transformation from PN to MPNST is challenging to diagnose due to difficulties in distinguishing cross-sectional imaging results and intralesional heterogeneity resulting in biopsy sampling errors.

**Methods and findings:**

This multi-institutional study from the National Cancer Institute and Washington University in St. Louis used fragment size analysis and ultra-low-pass whole genome sequencing (ULP-WGS) of plasma cell-free DNA (cfDNA) to distinguish between MPNST and PN in patients with NF1. Following in silico enrichment for short cfDNA fragments and copy number analysis to estimate the fraction of plasma cfDNA originating from tumor (tumor fraction), we developed a noninvasive classifier that differentiates MPNST from PN with 86% pretreatment accuracy (91% specificity, 75% sensitivity) and 89% accuracy on serial analysis (91% specificity, 83% sensitivity). Healthy controls without NF1 (participants = 16, plasma samples = 16), PN (participants = 23, plasma samples = 23), and MPNST (participants = 14, plasma samples = 46) cohorts showed significant differences in tumor fraction in plasma (*P* = 0.001) as well as cfDNA fragment length (*P* < 0.001) with MPNST samples harboring shorter fragments and being enriched for tumor-derived cfDNA relative to PN and healthy controls. No other covariates were significant on multivariate logistic regression. Mutational analysis demonstrated focal NF1 copy number loss in PN and MPNST patient plasma but not in healthy controls. Greater genomic instability including alterations associated with malignant transformation (focal copy number gains in chromosome arms 1q, 7p, 8q, 9q, and 17q; focal copy number losses in *SUZ12*, *SMARCA2*, *CDKN2A/B*, and chromosome arms 6p and 9p) was more prominently observed in MPNST plasma. Furthermore, the sum of longest tumor diameters (SLD) visualized by cross-sectional imaging correlated significantly with paired tumor fractions in plasma from MPNST patients (*r* = 0.39, *P* = 0.024). On serial analysis, tumor fraction levels in plasma dynamically correlated with treatment response to therapy and minimal residual disease (MRD) detection before relapse. Study limitations include a modest MPNST sample size despite accrual from 2 major referral centers for this rare malignancy, and lack of uniform treatment and imaging protocols representing a real-world cohort.

**Conclusions:**

Tumor fraction levels derived from cfDNA fragment size and copy number alteration analysis of plasma cfDNA using ULP-WGS significantly correlated with MPNST tumor burden, accurately distinguished MPNST from its benign PN precursor, and dynamically correlated with treatment response. In the future, our findings could form the basis for improved early cancer detection and monitoring in high-risk cancer-predisposed populations.

## Abstract 2

Jeffrey J. Szymanski and colleagues investigate the use of cell-free DNA ultra-low-pass whole genome sequencing to distinguish the malignant peripheral nerve sheath tumor (MPNST) from its benign precursor lesion in patients with Neurofibromatosis type 1 in United States.

## Author summary

**Why was this study done?:**

- Neurofibromatosis type 1 (NF1) is the most common inherited cancer predisposition syndrome.
- The leading cause of mortality in NF1 is malignant peripheral nerve sheath tumor (MPNST), an aggressive soft tissue sarcoma that arises from a benign plexiform neurofibroma (PN) precursor lesion.
- Transformation from PN to MPNST is challenging to detect by imaging (due to difficulty in distinguishing PN from MPNST radiologically) or by biopsy (due to intralesional heterogeneity), which often delays the diagnosis of MPNST and results in a worsened prognosis.

**What did the researchers do and find?:**

- We conducted a multi-institutional study involving 2 large NF1 referral centers, the National Cancer Institute and Washington University in St. Louis, involving 53 patients from whom plasma cell-free DNA (cfDNA) was analyzed using ultra-low-pass whole genome sequencing (ULP-WGS).
- We found that cfDNA from patients with MPNST harbors a shorter fragmentation profile compared to patients with PN or healthy donors. Using sequencing reads from this fragmentation profile, we quantified genome-wide copy number alterations (CNAs) in cfDNA and used CNAs to estimate the fraction of plasma cfDNA originating from tumor.
- Tumor fraction in plasma cfDNA distinguished pretreatment MPSNT from PN with 86% accuracy. Plasma cfDNA from MPNST and PN patients harbored focal copy number loss of NF1 not found in healthy donors. Strikingly, MPNST patient cfDNA also had significantly greater tumor genomic instability compared to PN, with CNAs in key genomic loci previously observed in MPNST tissue (i.e., gain of chromosome arm 8q and loss of 9p), which enabled sensitive and specific liquid biopsy discrimination of MPNST from PN.
- Plasma-derived tumor fraction correlated with tumor size from imaging in MPNST patients, and serial cfDNA analysis demonstrated the potential for noninvasive detection of minimal residual disease, treatment response assessment, and the potential for even greater assay sensitivity.

**What do these findings mean?:**

- Our findings suggest that cfDNA fragment analysis followed by ULP-WGS has the potential to be developed as a biomarker for treatment response and as a screening assay for early detection of MPNST.
- This study provides, to our knowledge, the first evidence for the ability of liquid biopsy to distinguish between benign and malignant tumors in a heritable cancer predisposition syndrome.

## Introduction

Neurofibromatosis type 1 (NF1) is an autosomal dominant disorder affecting one in 3,000 individuals worldwide and is caused by a heterozygous inactivating mutation in the tumor suppressor gene, *NF1*, located on chromosome 17q11.2 [1–3]. *NF1* encodes for the protein, neurofibromin 1, a negative regulator of the RAS signaling pathway. Thus, *NF1* loss-of-function mutations lead to hyperactivated RAS, whose downstream effects contribute to the elevated cancer risk in NF1 patients [4–6].

Approximately 50% of patients with NF1 develop histologically benign plexiform neurofibroma (PN) [1,7], in which Schwann cells acquire biallelic inactivation of the *NF1* gene [3,8]. Histologically, PNs are heterogeneous, consisting of primarily S100-positive Schwann cells (60% to 80%), as well as fibroblasts, endothelial cells, perineural cells, smooth muscle cells, mast cells, interspersed axons, and pericytes [2]. Imaging studies of PN mirror this heterogeneity, complicating the radiographic diagnosis of transformation to malignant peripheral nerve sheath tumor (MPNST), which occurs in 8% to 15% of patients with NF1 [1,9,10], as well as the accuracy of diagnostic tissue biopsy.

MPNST are aggressive cancers with a poor prognosis that frequently arise from within their benign PN precursors [9,11–13]. Due to rapid development of metastasis and resistance to both chemotherapy and radiotherapy, MPNST account for the majority of NF1-associated mortality [1,9] with a 5-year survival rate of only 20% [14]. Despite the high incidence and mortality of MPNST in the NF1 population, screening for malignant transformation and monitoring of MPNST is challenging. Clinical exam has poor sensitivity and may only signify MPNST when a PN lesion is showing sudden growth or causing severe pain [12,15]. Serial PN biopsies are impractical as 9% to 21% of NF1 patients will have multiple PN, with varying levels of malignant potential requiring surveillance [16–18]. Moreover, biopsies can yield false negative results due to geographic tumor heterogeneity resulting from MPNST arising from within heterogeneous PN precursor lesions [19]. Furthermore, standard cross-sectional imaging cannot distinguish MPNST from PN with adequate specificity [20,21]. Given the high prevalence of deadly MPNST in the context of a very common benign precursor lesion in a cancer-predisposed population, it is imperative that more reliable screening modalities be explored.

We and others have shown that other cancer types can be monitored through plasma cell-free DNA (cfDNA) analysis [22–25] and that malignancy can be associated with distinct cfDNA fragmentation profiles, typically characterized by shorter size [26–30]. We have also shown that sequenced MPNST tissue harbors broad chromosomal copy number alterations (CNAs) characteristic of increased genomic instability compared to PN, including in cases of MPNST transformation arising from within PN lesions [31,32]. Here, we hypothesize that this MPNST-intrinsic genomic instability is also detectable within plasma cfDNA and can be used to noninvasively distinguish MPNST from its benign precursor lesion.

In the current multi-institutional cross-sectional study involving 2 large referral centers for NF1 patients, the Washington University School of Medicine and the National Cancer Institute (NCI), we aimed to develop a noninvasive liquid biopsy method for distinguishing MPNST from its benign PN precursor using cfDNA fragmentomics and ultra-low-pass whole genome sequencing (ULP-WGS).

## Methods

### Study design

**Fig 1:**
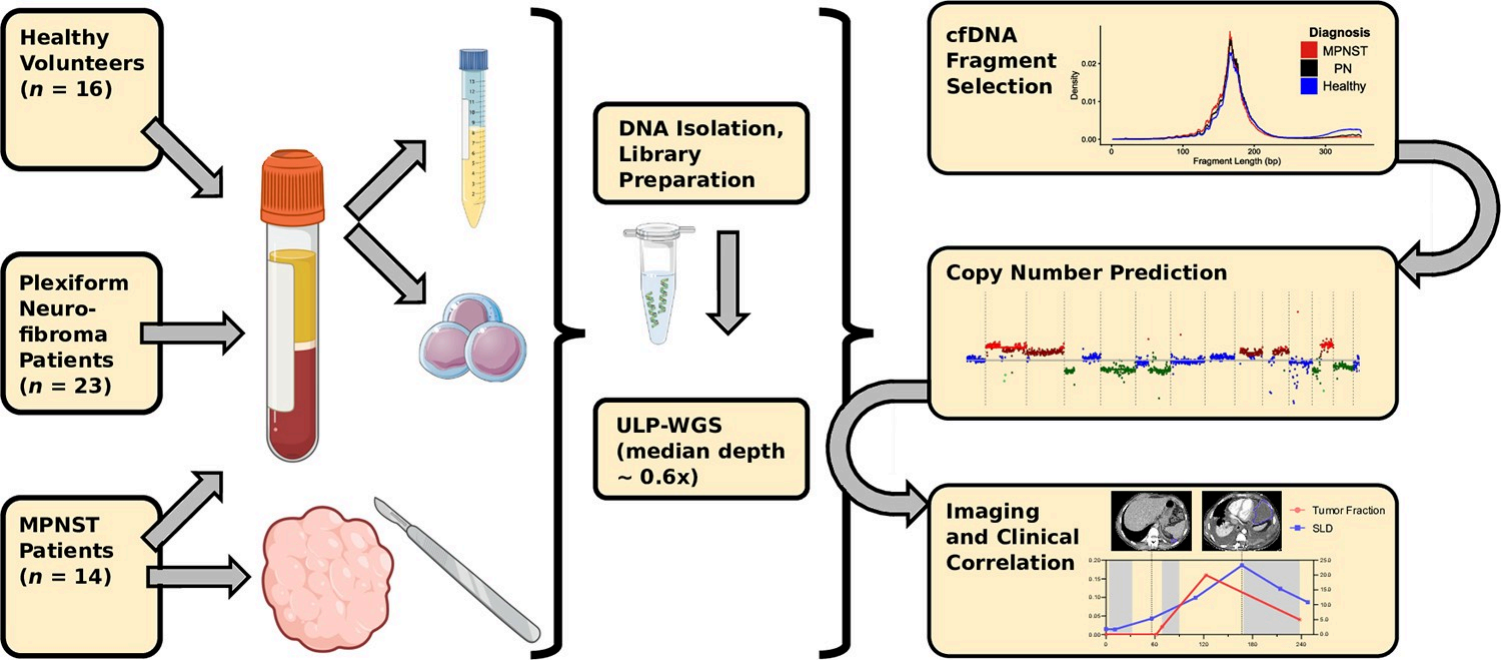
Study schema. Patients with imaging- and biopsy-proven MPNST and established PN along with healthy donors were enrolled onto this multi-institutional prospective cohort, with plasma collected for tumor fraction analysis at the time of study enrollment. Tumor fraction was assessed in all collected plasma samples by ULP-WGS followed by in silico size selection for short cfDNA fragments, which was used to train a noninvasive MPNST vs. PN classifier. During subsequent treatment and follow-up, MPNST patients underwent further serial imaging (analyzed by RECIST) and plasma sample collection (analyzed by ULP-WGS and in silico fragment size selection), with results correlated with each other and with clinical outcomes. cfDNA, cell-free DNA; MPNST, malignant peripheral nerve sheath tumor; PN, plexiform neurofibroma; RECIST, response evaluation criteria in solid tumors, version 1.1; SLD, sum of longest tumor diameters; ULP-WGS, ultra-low-pass whole genome sequencing.

This study used blood samples prospectively collected from NF1 patients with MPNST and PN tumors with the aim of distinguishing these different tumor types by plasma cfDNA analysis (**Fig 1**). Patients from the NCI and Washington University in St. Louis (WUSTL) with clinically and radiographically diagnosed PN or biopsy-proven MPNST were enrolled onto this multi-institutional cross-sectional study with written informed consent (NCI protocol NCT01109394, NIH Intramural IRB identifier 10C0086; NCI protocol NCT00924196, NIH Intramural IRB identifier 08C0079; WUSTL protocol NCT04354064, Washington University in St. Louis School of Medicine Human Research Protection Office IRB identifiers 201903142 and 201203042) between 2016 and 2020. NF1 status was determined clinically by consensus criteria [33]. Five participants with MPNST who did not meet NF1 consensus criteria were also included in the analysis. A total of 14 MPNST and 23 PN patients were enrolled with peripheral blood collected at the time of enrollment (**S1–S3 Tables**). MPNST patients had serial plasma samples collected for a total of 46 MPNST plasma samples (average 3, maximum 6 per participant, **S1 Table**). When available, tissue was also collected at a single time point (*n =* 4 participants). When peripheral blood mononuclear cells (PBMCs) were isolated from whole blood, these were sequenced as germline DNA (*n* = 16 participants). All patients underwent clinical management and follow-up by board-certified physicians per the standard-of-care. All samples were collected with informed consent for research and institutional review board approval in accordance with the Declaration of Helsinki. Protocols are available on ClinicalTrials.gov. A STROBE checklist was completed to ensure accurate and complete reporting of the study (see **Supplement**) [34].

### Healthy controls

After obtaining written consent, healthy donor blood samples were obtained at a single time point from appropriately consented donors at the NIH Department of Transfusion medicine (NIH protocol NCT00001846, NIH Intramural IRB identifier 99-CC-0168) and WUSTL Clinical Translational Research Unit (WUSTL protocol NCT04354064, Washington University in St. Louis School of Medicine Human Research Protection Office IRB identifiers 201903142 and 201203042) (**S4 Table**). Eligibility for healthy controls included age greater than 18 years old and no known history of neoplastic or hematological disorders. Protocols are available on ClinicalTrials.gov.

### Clinical specimens

After obtaining written informed consent for genomic analysis, serial peripheral blood samples were collected throughout the clinical course for consenting MPNST patients or at a single time point for PN patients and healthy controls. Treatment regimen for MPNST was determined by the primary treating clinicians and included radiotherapy, surgery, and cytotoxic chemotherapy (**S2 Table**).

Venous blood samples (10 to 30 mL) were collected in EDTA (BD Biosciences, San Jose, California) or Cell-Free DNA BCT (Streck Laboratories, La Vista, Nebraska) tubes. EDTA tubes were processed within 4 hours of collection, while Cell-Free DNA BCT tubes were processed within 7 days of collection. Whole blood samples were centrifuged at room temperature (NCI: 1,900 × *g* for 10 minutes, WUSTL: 1,200 × *g* for 10 minutes). Isolated plasma was centrifuged a second time at room temperature (NCI: 15,000 × *g* for 10 minutes, WUSTL: 1,800 × *g* for 5 minutes) in low-bind Eppendorf tubes to remove residual cells. Purified plasma was frozen at −80°C until cfDNA isolation.

### Plasma cell-free DNA isolation

Purified plasma was thawed at room temperature, and cfDNA was extracted from 2 to 8 mL of plasma using the QIAamp Circulating Nucleic Acid kit (Qiagen, Hilden, Germany). Extracted DNA concentration was measured using the Qubit dsDNA High-Sensitivity assay (ThermoFisher, Waltham, Massachusetts), and cfDNA concentration and quality were assessed using a Bioanalyzer (Agilent Technologies, Santa Clara, California) or Tapestation (Agilent Technologies, Santa Clara, California). Isolated cfDNA was stored at −20°C until library preparation.

### Germline DNA isolation and processing

After centrifuging clinical venous blood samples and removing plasma supernatant per above, the red blood cells and buffy coat were resuspended in PBS for germline DNA extraction using the DNeasy Blood and Tissue kit (Qiagen, Hilden, Germany). For a subset of samples, germline DNA from PBMCs was collected in and extracted using PAXgene Blood DNA tubes and kit (PreAnalytix, Germantown, Maryland). DNA was stored at −20°C until further processing. Germline DNA was then fragmented using a LE220 focused ultrasonicator (Covaris, Woburn, Massachusetts) or a Q800R3 sonicator (Qsonica LLC, Newtown, Connecticut) according to the manufacturer’s instructions and previously published methods [35] to a target length of 200 bp. DNA lengths were assessed using a Bioanalyzer (Agilent Technologies, Santa Clara, California).

### Tumor DNA isolation and processing

Tumor tissue was not procured for research unless clinically indicated and available following the standard clinical pathology workflow. When available, tumor tissue was snap-frozen and stored at −80°C or stored in formalin-fixed paraffin-embedding (FFPE). Nucleic acids were isolated from tumor FFPE samples using the manufacturer’s protocol with the AllPrep DNA/RNA FFPE kit (Qiagen, Hilden, Germany). DNA was extracted from snap-frozen tumor tissue samples using the DNeasy Blood and Tissue kit (Qiagen, Hilden, Germany). Extracted DNA was stored at −20°C until further processing. Tissue DNA was subsequently fragmented using a LE220 focused ultrasonicator (Covaris, Woburn, Massachusetts) or Q800R3 sonicator (Qsonica LLC, Newtown, Connecticut) and analyzed using a Bioanalyzer (Agilent Technologies, Santa Clara, California) as described above.

### DNA library construction and sequencing

Sequencing libraries were constructed from cfDNA (NCI 5 to 15 ng, WUSTL 10 to 60 ng) or germline/tumor DNA (NCI 100 ng, WUSTL 32 ng) using commercial kits per the manufacturers’ instructions: TruSeq Nano (Illumina, San Diego, California) for NCI samples and Kapa HyperPrep (Roche, Basel, Switzerland) for WUSTL samples. Constructed libraries were balanced, pooled, and sequenced using 150 bp paired-end reads on a NovaSeq (Illumina, San Diego, California) or HiSeq 4000 (Illumina, San Diego, California). Data were then quality filtered and pooled for analysis.

### Copy number alteration and tumor fraction analysis

Sequencing data were demultiplexed, and raw reads were quality filtered using fastp v.0.2. Quality-filtered reads were then aligned to the hg19 human genome assembly using BWA v.0.7.17. Aligned reads were deduplicated with Samtools v.1.7, then downsampled to 10 million read pairs (WGS coverage approximately 0.6×), or separately for comparison purposes to 5 million read pairs (WGS coverage approximately 0.3×). Genomic coverage was estimated using MosDepth [36]. To enrich for circulating tumor DNA (ctDNA) fragments, in silico size selection was applied to all cfDNA samples [28]. Only quality-filtered reads between fragment lengths of 90 and 150 bp were considered for copy number and tumor fraction analysis for cfDNA samples, while such size selection was not performed for tumor and germline samples. GC content and mappability bias correction, depth-based local copy number estimates, and copy number–based estimation of tumor fraction were then performed using the ichorCNA tool (Broad v.0.2.0) [37]. Briefly, reads were summed in nonoverlapping windows of 10^6^ bases; local read depth was corrected for GC bias and known regions of low mappability, and artifacts were removed by comparison to ichorCNA’s built-in healthy control reference. CNAs were predicted using recommended low tumor fraction parameters for cfDNA samples and default parameters for tumor and germline samples. X and Y chromosomes were not considered in copy number ratios. ichorCNA then used these binned, bias-corrected copy number values to model a two-component mixture of tumor-derived and nontumor-derived fragments, from which it inferred the fraction of reads in each sample originating from tumor (tumor fraction) [37]. Visualization of genome-wide CNAs at specific loci (**Fig 3**) was generated from compiled log_2_ ratios of copy number for all study plasma specimens (*n =* 85 samples). Reads were classified as copy number gain if log_2_ of the copy number ratio was >0.58 (log_2_ (3/2)) and loss if log_2_ of the copy number ratio was <−1.0 (log_2_ (1/2)). Bin CNA plots (**S1 Fig**) reflect copy number changes from baseline in the tumor-only fraction of each sample. Both copy number state and tumor fraction were determined by ichorCNA [37].

### Fragment size analysis

Following the sequencing quality control, deduplication, alignment, and downsampling steps described above, read-pair fragment sizes for cfDNA samples were calculated using deepTools bamPEFragmentSize [38]. The distribution of each sample’s fragment sizes was estimated by kernel density. cfDNA fragment size distributions were compared between the 3 clinical states (healthy control, PN, and MPNST) and between high and low tumor fraction samples by two-sided Kolmogorov–Smirnov testing.

### Comparisons of cfDNA tumor fraction to imaging

Patients with MPNST and PN were monitored by CT, MRI, and/or FDG-PET imaging at the treating institution at the managing clinicians’ discretion. For patients with MPNST, radiographic tumor burden was quantified by sum of the longest tumor diameters (SLD) per RECIST 1.1 criteria [39]. For comparison to serial time point cfDNA tumor fractions, each plasma sample was matched to the nearest SLD value at the primary institution within 30 days and without any interceding change of therapy. SLDs and plasma tumor fraction levels were then assessed using Pearson correlation coefficient. For comparisons of plasma tumor fraction to clinical status by RECIST, tumor fraction values were first normalized per patient to the lowest value detected on serial analysis, and then log_2_ transformed to generate the final plotted values in **Fig 5B**. Changes in clinical status were assessed and categorized as complete response, partial response, stable disease, or progressive disease per RECIST 1.1 criteria [39]. RECIST 1.1 scoring was performed on serial imaging studies relative to a patient’s baseline scan.

### Power and statistical analyses

Previous tissue-based studies have shown that PN harbor few genome-wide CNAs [40,41] but acquire significant genomic instability during malignant transformation to MPNST [32,41,42]. Based on these known significant CNA differences between MPNST and PN tumors, we assumed a large effect size would also be evident comparing MPNST plasma tumor fraction to plasma from PN patients or healthy controls. Using Cohen’s *f* = 0.6 with an α = 0.05 and power = 0.80, we projected that the sample size needed to detect differences between these 3 categories would be *n =* 10 per group. Our category group sizes met or exceeded this estimate for all comparisons (**S1–S4 Tables**).

When testing associations between plasma tumor fraction and clinical status (**Fig 4**), we limited MPNST plasma samples to those collected either prior to all treatments or after a washout period of at least 21 days after completion of chemotherapy or radiotherapy (designated as pretreatment or baseline MPNST samples below). The distributions of plasma tumor fraction for each clinical status were compared by Kruskal–Wallis H test with pairwise comparisons by Dunn test. To further compare pretreatment MPNST to benign PN patients, we generated a receiver operating characteristic (ROC) curve of plasma tumor fraction. Tumor fraction values derived from ctDNA-enriched 90 to 150 bp fragments were compared to tumor fractions derived from all cfDNA fragment lengths. For ctDNA-enriched tumor fraction, an optimized cutpoint was determined by Youden’s index (the point on the ROC curve that maximizes sensitivity + specificity– 1), and high and low plasma tumor fraction groups by cutpoint were compared to clinical status by Fisher exact test. A logistic regression was also performed for the MPNST versus PN groups using the glm function in *R*, evaluating the effects of age, sex, and institution in addition to pretreatment plasma tumor fraction. Leave-one-out cross-validation was performed in *R* using the caret package. The reverse Kaplan–Meier method was used to estimate follow-up times. Statistical analyses were performed using *R* v.3.6.1 or Prism 9 (GraphPad Software).

## Results

### Overview and patient characteristics

The primary objective of this study was to noninvasively differentiate MPNST from benign PN by analyzing and quantifying genomic CNAs in blood plasma cfDNA (**Fig 1**). To quantify CNAs, we profiled 105 biospecimens including 85 plasma samples from 53 participants by ULP-WGS downsampled to 10 million paired reads (approximately 0.6× genome-wide coverage) (**Fig 1, S1 Table**). Participant groups compared were MPNST and PN patients as well as healthy donor controls. Specimen types included blood plasma cfDNA, blood leukocyte germline DNA, and frozen tumor specimens (**S1 Table**). The median age was 36, 27, and 32.5 for MPNST patients, PN patients, and healthy donors, respectively (**S2–S4 Tables**). Exclusion criteria included diagnosis of a non-MPNST malignancy. No PN patients developed any clinical or radiographic evidence of MPNST transformation during a median study follow-up time of nearly 2 years (median 690 days; interquartile range (IQR) of 531 to 1,140 days; **S3 Table**). For 2 participants, sar015 and sar037, large PN resection was performed for lesion-related morbidity with final pathology confirming the PN diagnosis. Among patients with MPNST, 86.7% received chemotherapy, 35.7% received radiotherapy, and 42.9% underwent surgical resection (**S2 Table**).

### Genome-wide CNAs from tumor are detected in plasma

**Fig 2:**
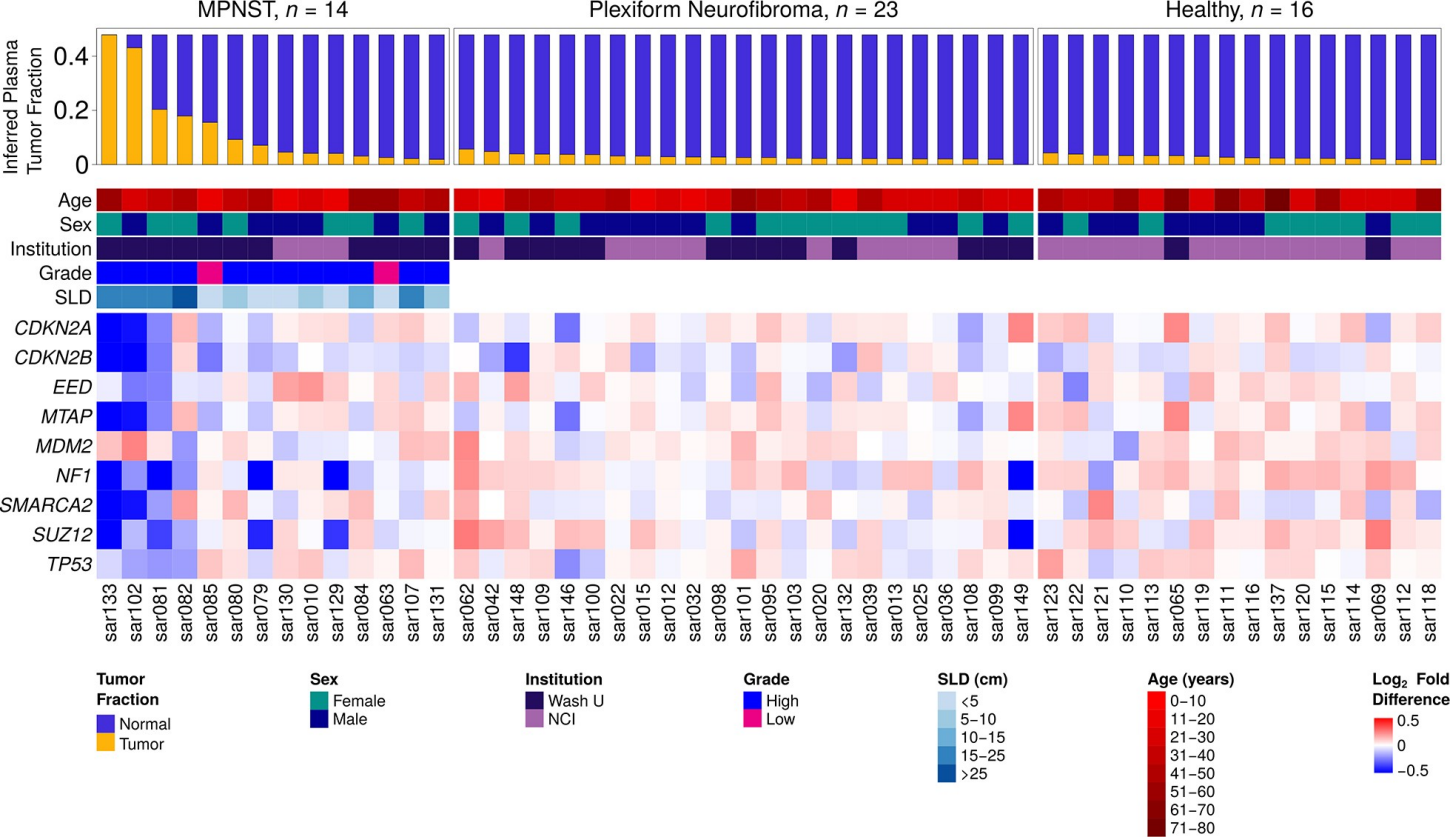
Participant characteristics and CNAs. Heatmap includes all 53 participants in this study, categorized by diagnosis. Each column represents one study participant with ID labels shown below. Highest tumor fraction in plasma and important tumor and participant characteristics are displayed in the top panel. The lower panel shows CNAs in genes relevant to NF1 and MPNST pathogenesis, depicted as log_2_ of copy number ratio. CNA, copy number alteration; MPNST, malignant peripheral nerve sheath tumor; NF1, neurofibromatosis type 1; SLD, sum of longest tumor diameters as determined by RECIST 1.1 criteria.

**Fig 3:**
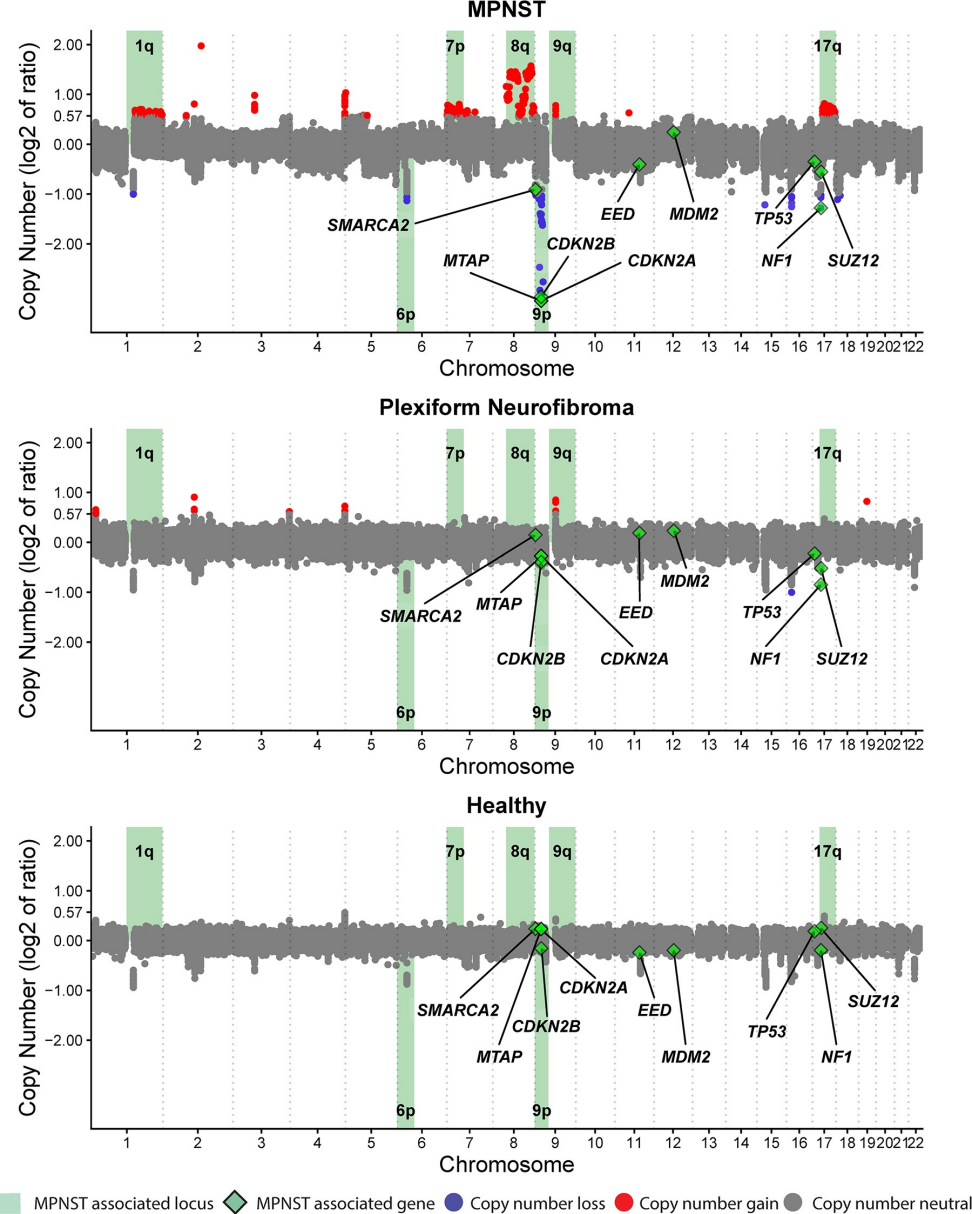
Aggregate CNAs measured across the genome. Plots represent plasma cfDNA data compiled from all blood plasma specimens from (A) MPNST (*n =* 46), (B) PN (*n* = 23), or (C) healthy donors (*n* = 16) in this study. Copy number ratios across the genome are shown on a log_2_ scale with significant gains in red, significant losses in blue, and regions without significant gain or loss depicted in gray (**Methods**). Loci highlighted in green have been previously associated with MPNST or NF1, with associated genes also labeled and depicted by green diamonds. cfDNA, cell-free DNA; CNA, copy number alteration; MPNST, malignant peripheral nerve sheath tumor; PN, plexiform neurofibroma.

Approximately 86% of MPNST and PN patients enrolled onto our study met the NIH criteria for NF1 diagnosis. There was no difference in tumor fraction between the MPNST patients who met NIH criteria and those who did not (*P* = 0.93 by Wilcoxon rank-sum test). Genomic copy number analysis of plasma cfDNA revealed that focal somatic CNAs that have previously been associated with PN tumor progression in NF1 patients [32] were prominently observed in MPNST patients and were occasionally found in PN patients, but absent in healthy controls (**Figs 2 and 3**). For example, focal loss of *CDKN2A/B*, *MTAP*, *SMARCA2*, and *SUZ12*, alterations shown to be associated with malignant transformation of PN [32,43,44], was observed in plasma from MPNST patients. *CDKN2B and SUZ12* losses were also found in 2 PN patients, while *SMARCA2* appeared to be copy number neutral in plasma across the full PN cohort. Loss of *SUZ12* correlated with *NF1* loss, consistent with both genes’ location in the 17.q11.2 genomic locus [32,45]. Additionally, we observed broader copy number gains in chromosome arms 1q, 7p, 8q, 9q, and 17q as well as broad losses in arms 6p and 9p only in MPNST patient plasma, again consistent with previous findings from NF1 MPNST tumors [32,42,46] (**Fig 3**). Finally, while many types of *NF1* gene activation can underlie the NF1 disease process, we observed evidence of one of these, *NF1* copy number loss, only within MPNST and PN patients, but not in healthy donor controls.

Given the observed copy number changes in patient plasma, we next compared genome-wide CNAs and associated tumor fractions across specimen types. For MPNST cases where tumor, leukocyte, and plasma were all available, the observed copy number aberrations were most prominent in the tumor samples, but also detected in plasma cfDNA prior to treatment, with a pattern reflective of the original tumor (**S1 Fig**). The magnitude of these CNAs decreased in posttreatment cfDNA compared to pretreatment cfDNA, and germline samples harbored the least detectable CNAs. This trend also held for estimated tumor fractions, representing a sample’s aggregate genome-wide copy number changes. As expected, there was no such increase in tumor fraction observed in PN lesions or in cfDNA derived from PN or healthy adults.

### Plasma tumor fraction distinguishes MPNST from plexiform neurofibroma

**Fig 4:**
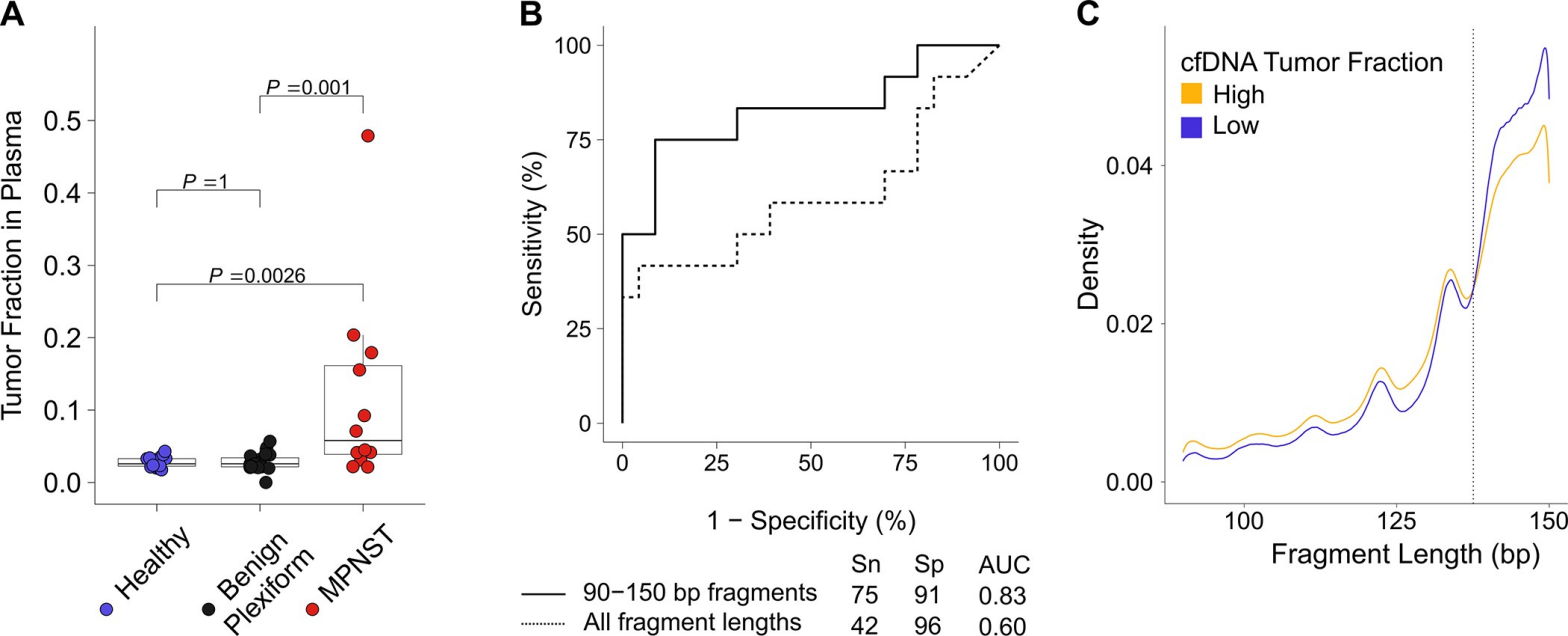
Tumor fraction in plasma stratifies patients by diagnosis. **(A)** Tumor fraction in participants with available pretreatment plasma cfDNA (*n =* 53), stratified by clinical diagnosis, with significance assessed by the Dunn test after Kruskal–Wallis analysis of variance. **(B)** ROC curve of plasma cfDNA tumor fraction comparing pretreatment MPNST to PN patients. Solid line represents tumor fraction data derived only from 90–150 bp fragments, while dotted line represents tumor fractions derived from all fragment lengths. Confusion matrix is reported separately (**S5 Table**). **(C)** Fragment length density for cfDNA in MPNST and PN patients (*n* = 69 samples) with high (>0.0413) versus low (<0.0413) tumor fractions in plasma as determined by the Youden’s index-optimized cutpoint from the ROC curve. The dashed line highlights an inflection in the curves with high tumor fraction samples enriched for shorter cfDNA fragment sizes (<138 bp) and low tumor fraction samples enriched for longer cfDNA fragment sizes (*D* = 0.078, *P* < 0.001 by two-sample Kolmogorov–Smirnov test). Data are shown for sequencing reads within the 90 to 150 bp in silico size-selection range (**Methods**). AUC, area under the curve; bp, base pairs; cfDNA, cell-free DNA; MPNST, malignant peripheral nerve sheath tumor; PN, plexiform neurofibroma; ROC, receiver operating characteristic; Sn, sensitivity; Sp, specificity.

Given that tumor-derived CNAs were detected in plasma cfDNA from MPNST patients, we next investigated the ability of plasma tumor fraction, inferred from the genome-wide copy number data following in silico size selection of 90 to 150 bp fragments, to noninvasively differentiate MPNST from PN. Plasma tumor fraction was compared between healthy controls, PN, and all pretreatment MPNST samples. Strikingly, baseline cfDNA tumor fraction differentiated MPNST from both healthy (*P* = 0.0026) and PN (*P* = 0.001) participants. PN and healthy donors did not differ in cfDNA tumor fraction (*P* = 1) (**Fig 4A**). Median tumor fraction levels in healthy (0.026) and PN (0.026) groups were lower than in MPNST (0.058). Comparing plasma tumor fractions between pretreatment MPNST patients and PN patients, ROC analysis further demonstrated an area under the curve (AUC) of 0.83 (**Fig 4B**), which was higher with approximately 0.6× ULP-WGS than approximately 0.3× (**Methods; S4 Fig**). Notably, AUC decreased to 0.60 when considering all cfDNA fragment sizes, highlighting the importance of our in silico size selection step. This signified the ability to accurately discriminate between MPNST and PN using only plasma tumor fraction levels derived from ULP-WGS followed by in silico size selection.

Thus, utilizing a Youden’s index-optimized cutpoint of 0.041, pretreatment plasma tumor fraction differentiated MPNST from PN with an area under the ROC curve of 0.83, and sensitivity of 75% and specificity of 91%, with 21 of 23 PN cases successfully classified based on pretreatment plasma tumor fraction alone (*P* = 0.001) (**S5 Table**). This compared favorably to reports of other diagnostic modalities including MRI features and image-guided core-needle biopsy (**S6 Table**). Model performance was retained in leave-one-out cross-validation using a penalized regression model where overall accuracy was 75% (95% CI 66% to 83%) and improved to 89% with AUC of 0.89, Youden’s index-optimized sensitivity of 83%, and specificity of 91% when considering the highest plasma tumor fraction measured per participant on serial time point analysis (**S7 Table**). In a multivariate binary logistic regression model including age, sex, and institution, baseline plasma tumor fraction remained significantly associated with clinical status (*P* = 0.04), while the other covariates were not (**S8 Table**).

Validating our original fragment size selection strategy, we observed fragment size differences between clinical states as defined by the tumor fraction ROC cutpoint. Using high-tumor fraction versus low-tumor fraction groups determined by the optimal cutpoint of 0.041, there was a significant difference in fragment length distributions (*D* = 0.078, *P* < 0.001 by two-sample Kolmogorov–Smirnov test) with high-plasma tumor fraction cases enriched for shorter cfDNA fragments and low-plasma tumor fraction cfDNA enriched for longer fragments (**Fig 4C**). Similarly, clinically classified MPNST patients harbored significantly shorter cfDNA fragments compared to PN patients (*D* = 0.032, *P* < 0.001) and healthy donors (**S2 Fig**). Thus, fragmentation profiles appear unique in patients with MPNST compared to those with benign PN.

### MPNST plasma tumor fraction correlates with disease burden by imaging

**Fig 5:**
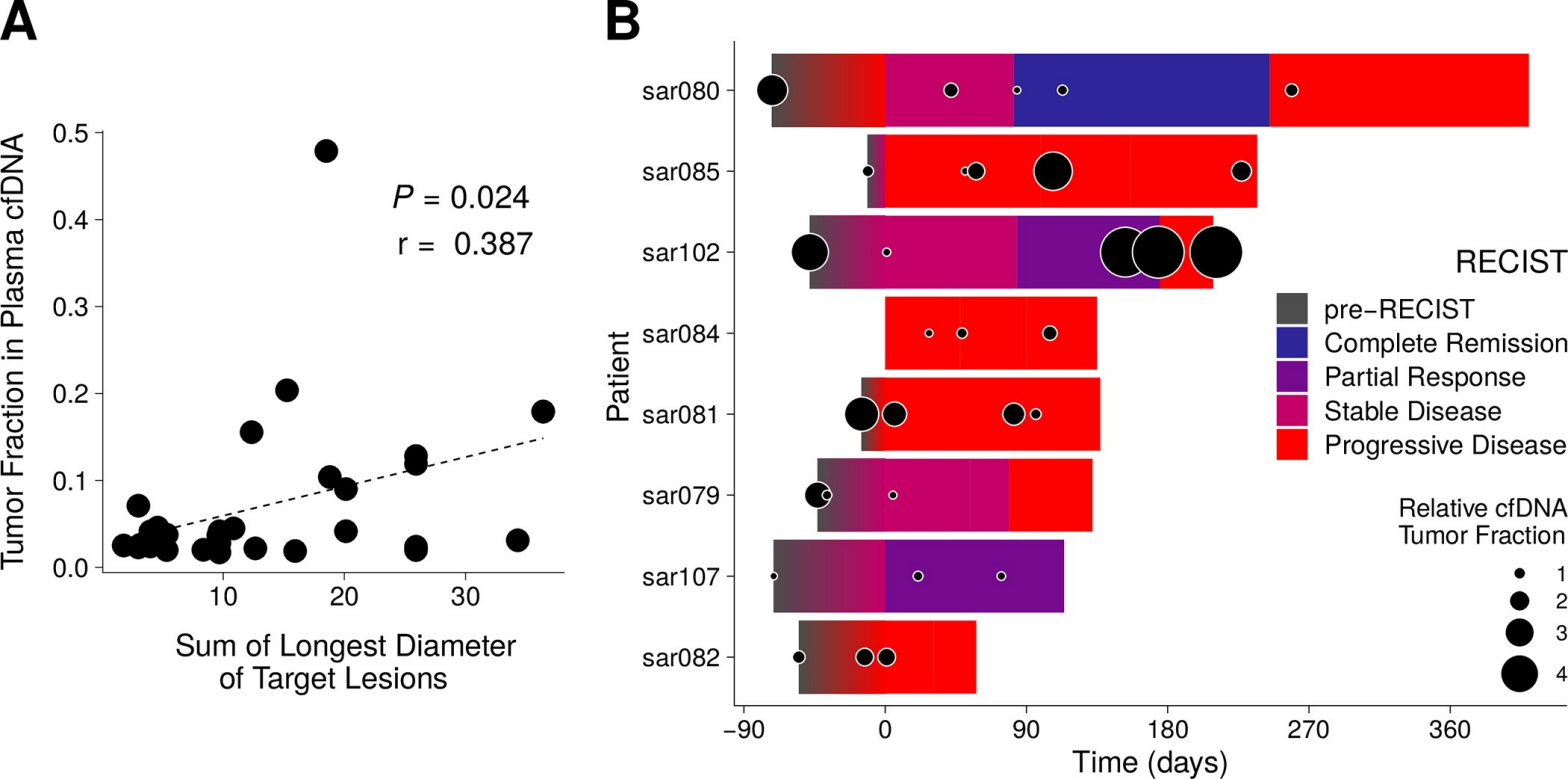
Tumor fraction in plasma correlates with imaging. **(A)** SLD of target lesions for all MPNST patients are plotted against each SLD’s temporally closest plasma tumor fraction value (**Methods**). Pearson correlation coefficient is significant at *P* = 0.024. **(B)** Timelines of RECIST classification for MPNST patients that underwent serial monitoring (*n =* 8; **S3 Fig**). Overlaid are cfDNA tumor fraction levels, normalized per patient to the lowest value detected on serial analysis, and then log_2_ transformed (**Methods**). cfDNA, cell-free DNA; MPNST, malignant peripheral nerve sheath tumor; RECIST, response evaluation criteria in solid tumors, version 1.1; SLD, sum of longest tumor diameters.

Having established plasma cfDNA fragment size and tumor fraction as a specific means to classify MPNST cases noninvasively, we next investigated the relationship between plasma tumor fraction derived using our assay and radiologically measured tumor burden. Radiographic tumor burden was quantified by the sum of longest tumor diameters (SLD) by RECIST 1.1 criteria [39] and compared to matched plasma cfDNA tumor fraction levels (**Methods**). A significantly positive correlation was observed between SLD and plasma tumor fraction (Pearson *r* = 0.387, *P* = 0.024) (**Fig 5A**). Because RECIST SLD measurements are restricted to 5 total lesions, 2 lesions per organ and do not include bony disease, SLD may underestimate tumor burden in metastatic MPNST patients [39]. Conversely, SLD may overestimate the size of malignant tissue in primary MPNST lesions, which often arise from within PN, with the relative contribution of PN versus MPNST tissue to the overall lesion size difficult to accurately assess radiographically [47]. These challenges limit the ability of SLD to accurately define MPNST disease burden and may explain why the correlation of SLD to tumor fraction was not stronger.

**Fig 6:**
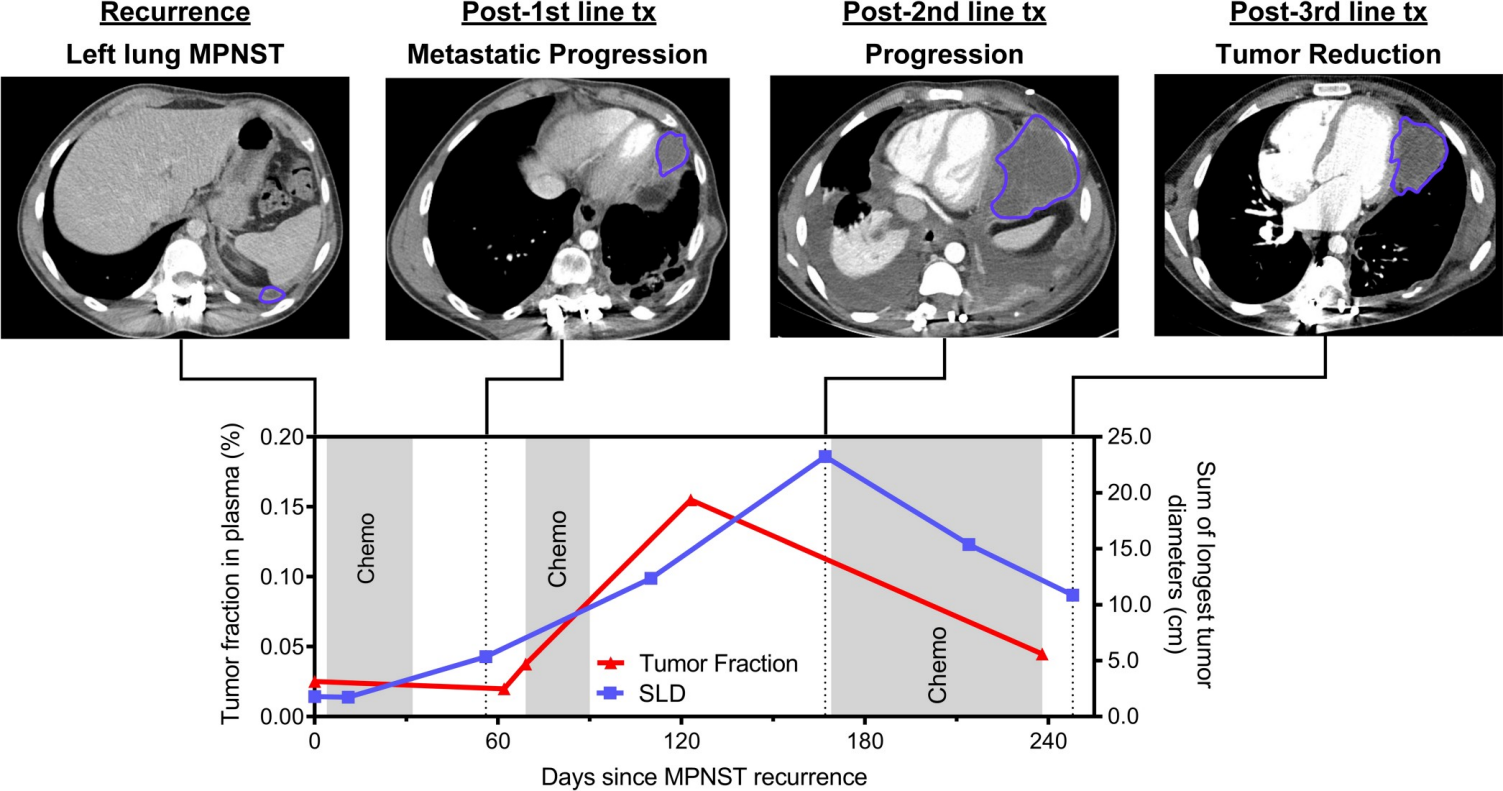
Monitoring tumor burden vignette (sar085). This patient had a high-grade thoracic MPNST recurrence that progressed rapidly through first- and second-line chemotherapy but responded to third-line chemotherapy. Tumor fraction in plasma was initially undetectable, then rapidly increased during first- and second-line chemotherapy, followed by a rapid decrease during third-line chemotherapy. This dynamic tumor fraction in plasma correlated well with the SLD measured radiographically by RECIST 1.1 criteria. Chemo, chemotherapy; cm, centimeters; MPNST, malignant peripheral nerve sheath tumor; SLD, sum of longest tumor diameters; Tx, treatment.

**Fig 7:**
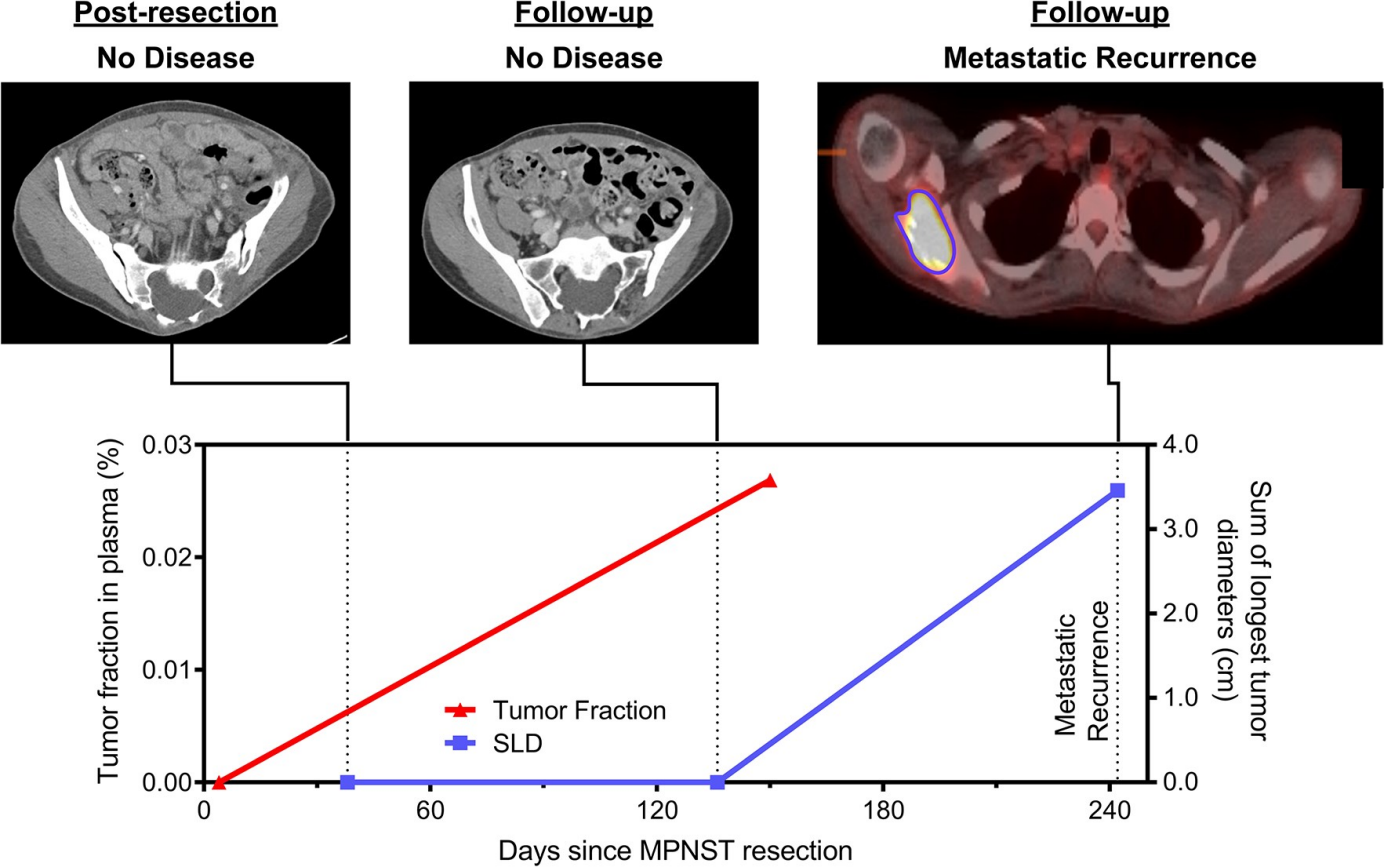
Early detection of relapse vignette (sar080). This patient previously had a high-grade pelvic MPNST that was completely resected with no evidence of residual disease after surgery. Tumor fraction in plasma was undetectable following resection but was detected 150 days later (tumor fraction = 0.021); this preceded metastatic recurrence identified on surveillance imaging by 89 days. cm, centimeters; MPNST, malignant peripheral nerve sheath tumor; SLD, sum of longest tumor diameters as determined by RECIST 1.1 criteria.

**Fig 8:**
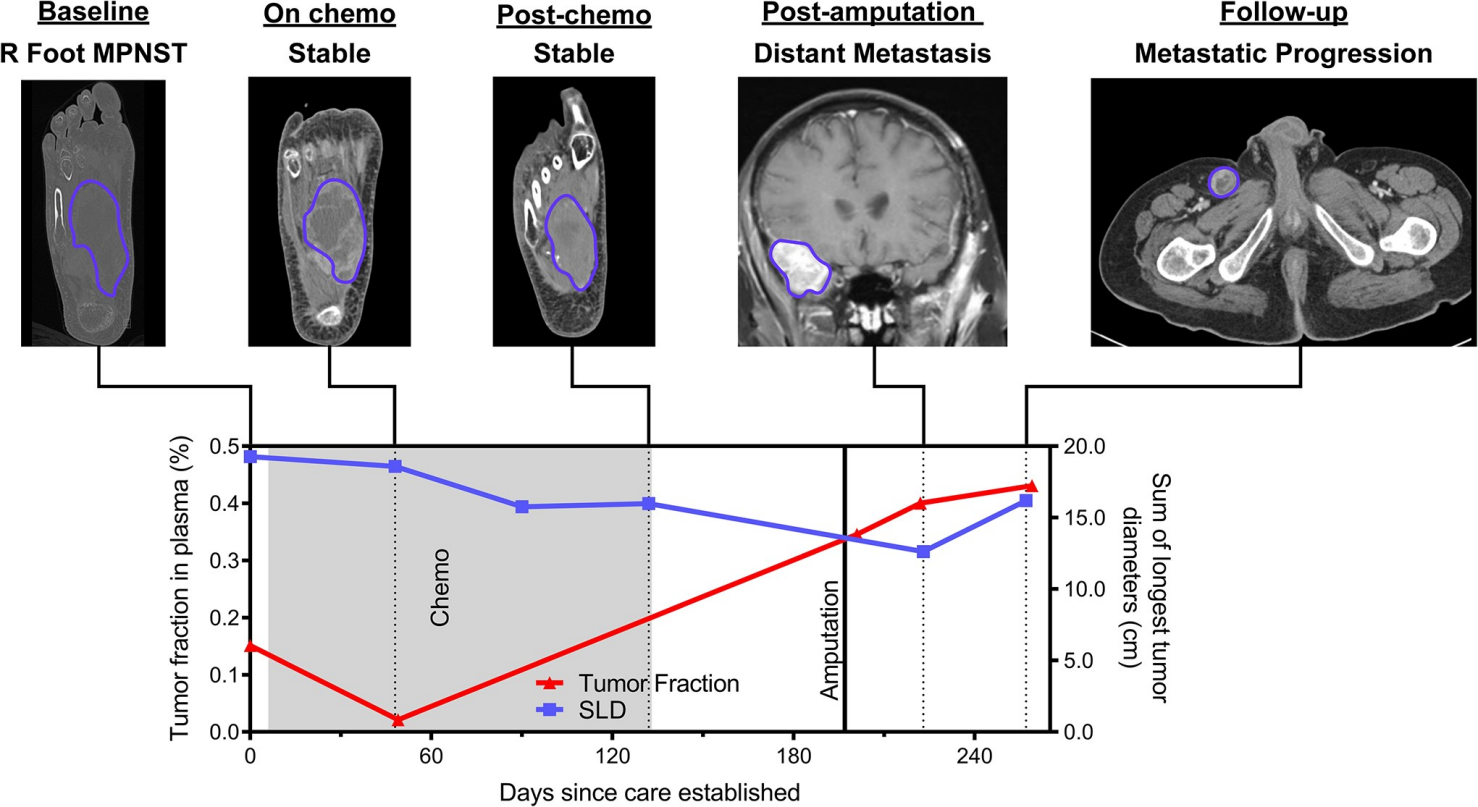
Clinical decision-making vignette (sar102). This patient had a high-grade MPNST in the right foot that was stable by imaging and therefore had chemotherapeutic agents held in order to undergo an elective lower limb amputation for improved quality of life. Tumor fraction in plasma, however, increased during this presurgical time period, consistent with progressive metastatic disease, which became apparent on imaging shortly after surgery. The patient would not have had chemotherapy held to undergo lower limb amputation had there been earlier evidence of progressive metastatic disease. Chemo, chemotherapy; cm, centimeters; MPNST, malignant peripheral nerve sheath tumor; SLD, sum of longest tumor diameters as determined by RECIST 1.1 criteria.

We furthermore dynamically tracked both SLD and plasma tumor fractions over time in patients with serial time point data (**S1 Table**). We applied RECIST 1.1 criteria [39] in these patients to classify radiographic response to therapy (**Methods**). Dynamic changes in plasma tumor fraction typically correlated with but preceded imaging changes (**Fig 5B, S3 Fig**). We thus studied timelines of disease progression versus response for MPNST patients, comparing imaging SLD to plasma tumor fraction in the context of the specific treatments received (**Figs 6–8, S3 Fig**). For example, sar085 presented with a small recurrent lung MPNST that rapidly progressed into multiple thoracic metastases despite 2 lines of cytotoxic chemotherapy, but then partially responded to third-line chemotherapy (**Fig 6**). Interestingly, plasma tumor fraction levels closely tracked with SLD from CT imaging throughout this treatment course. These data suggest that plasma tumor fraction could thus be utilized to monitor treatment response.

There were also several instances where we observed cfDNA tumor fraction elevations anticipating and preceding corresponding SLD increases. sar080 is an illustrative example in which the patient had complete resection of a right pelvic MPNST prior to plasma cfDNA analysis (**Fig 7**). Initial plasma tumor fraction was not detected, which was consistent with our finding of no evidence of disease by imaging. Follow-up imaging again showed no evidence of disease 2.5 months later. Plasma tumor fraction rose, however, just 14 days later and preceded radiographic recurrence by 89 days, suggesting that the measurement of plasma tumor fraction could be utilized as a sensitive surveillance tool for minimal residual disease (MRD) detection following the completion of therapy.

sar102 illustrates another example where cfDNA tumor fraction elevation preceded radiographic progression. This patient had metastatic MPNST with a right foot primary tumor (**Fig 8**). During and following over 4 months of cytotoxic chemotherapy, imaging showed a partial response with persistently decreasing SLD. Given radiographic evidence of disease control, the medical team then decided to hold cytotoxic chemotherapy for an elective lower limb amputation with the goal of improving quality of life (pain reduction and ability to use a prosthetic limb). In contrast to SLD, however, plasma tumor fraction increased 14-fold during this same interval (**S1 Table**). Following cessation of cytotoxic agents and amputation, the patient was found to have significant widespread tumor progression with the development of multiple distant metastases including brain metastases. Here, plasma tumor fraction was more consistent than serial imaging with this patient’s ultimate clinical status and could have influenced the decision to hold chemotherapy in order to perform an elective, palliative amputation. This illustrates the potential utility of plasma cfDNA tumor fraction analysis for dynamically monitoring disease, anticipating progression, and influencing clinical decision-making.

## Discussion

In this multi-institutional cohort study, we performed fragment size analysis and ULP-WGS of cfDNA to noninvasively detect genome-wide CNAs and derive tumor fraction in plasma, which we used to differentiate between MPNST and PN patients. To our knowledge, this is the first demonstration of liquid biopsy to distinguish between malignant and premalignant solid tumor in the setting of a cancer predisposition syndrome. Specifically, we observed that patients with MPNST harbor a unique cfDNA fragmentation profile and have significantly greater tumor genomic instability evident in plasma compared to PN patients. We also demonstrated that cfDNA analysis can be used to dynamically track treatment response in MPNST patients, potentially with greater precision than standard cross-sectional imaging.

MPNST are aggressive soft tissue sarcomas that can be difficult to distinguish from their benign precursors, illustrating the need for new testing modalities for better disease detection and surveillance. Our data illustrate several important points. First, we accurately identified copy number–altered genomic loci characteristic of malignant transformation from PN using ULP-WGS of cfDNA (**Figs 2 and 3**) [32,43]. Specifically, loss of *CDKN2A/B*, *SMARCA2*, and *SUZ12* were commonly found only in MPNST plasma samples, while loss of *NF1* was observed in both MPNST and PN plasma, but not healthy controls. When paired tumor was available, plasma cfDNA-detected CNAs recapitulated tissue patterns of genomic instability (**S1 Fig**). Together, these data suggest that even at low sequencing coverage, the genomic features of both NF1 and of progression from PN to MPNST are detectable in affected patients’ plasma.

We strikingly also show that cfDNA tumor fraction derived from genome-wide CNAs after selecting for shorter fragment lengths, without applying prior knowledge of patient-specific mutational profiles, differentiated MPNST from PN with high specificity (91%) and moderate sensitivity (75%) pretreatment (**Fig 4B**) and both high specificity (91%) and sensitivity (83%) when measured serially (**S7 Table**). This strongly suggests that cfDNA tumor fraction could be a valuable adjunct to aid in monitoring patients with PN with the goal of early cancer detection. Currently, malignant transformation in NF1 patients is difficult to screen for due to overlapping clinical symptoms and radiographic findings that are also associated with benign PN [48,49]. Reflective of this, current standard practice for PN surveillance is to obtain imaging only when clinically indicated. Moreover, clinical surveillance for symptoms such as lesion-associated pain have a low specificity for identifying MPNST on subsequent workup [48,50,51]. Furthermore, the lack of reliable radiographic characteristics using standard sequences that can be replicated across institutions has contributed to overall limited sensitivity of MRI (62.5% to 84%) and specificity of FDG-PET (52.2% to 83%) for MPNST detection [20,21,52].

Still, it will be important to fully consider state-of-the-art imaging results, such as anatomic MRI features [53], and integrate them with plasma cfDNA results in the future in order to maximize combined modality performance, which we suggest may be possible by coupling advanced MRI features with our highly specific liquid biopsy assay (**S6 Table**). Indeed, in current practice, confirmation of MPNST identified by clinical/imaging suspicion is usually attempted by solid tumor biopsy, which can be exquisitely painful given the peripheral nerve site, is often technically difficult due to lesions’ propensity for localizing to viscera and the retroperitoneum, and is associated with serious complications including nerve palsy and dissemination of malignant tumor cells [53]. Additionally, biopsy is not without diagnostic caveat, as the development of MPNST from within PN lesions causes sampling bias with image-guided biopsies shown to result in low negative predictive value (NPV), with 50% of NF1 patients diagnosed with PN on image-guided core-needle biopsy being subsequently reclassified as MPNST following surgical resection in one retrospective study [19]. Unlike image-guided tumor biopsy, our liquid biopsy approach to measure tumor fraction reflects chromosomal instability throughout the body, thus limiting the potential for sampling bias.

Previous studies have demonstrated the ability of liquid biopsy testing to distinguish patients with cancer from healthy individuals [26,54–57]. Here, we significantly extend this body of work to show that a liquid biopsy test can detect malignant transformation in the context of hereditary cancer predisposition, distinguishing patients with a malignant solid tumor from those with its benign precursor lesion. We also build upon prior literature showing that fragmentation profiles and lengths of cfDNA from cancer patients are distinct from those in healthy donors [26,28–30], showing for the first time that malignancy-associated cfDNA is significantly shorter than its benign-associated counterpart (**Fig 4C, S2 Fig**). We additionally demonstrate that increasing sequencing coverage to approximately 0.6× improves the sensitivity of our ULP-WGS-based assay (**S4 Fig**).

We further note that the moderate pretreatment sensitivity of our cfDNA technology for detecting MPNST versus PN, which we showed was 75% with 91% specificity, compares favorably to landmark early cancer detection studies such as the ones from Grail [56], Thrive [55], and Stanford [54], which reported sensitivities of approximately 20% to 50% with approximately 95% to 99% specificity for common stage 1 to 2 solid tumor malignancies. Indeed, when we set the specificity of our assay to 100%, we still achieved a comparable sensitivity of 50% pretreatment and 58% when considering the highest tumor fraction on serial time point analysis (**S7 Table**). Overall, our plasma cfDNA assay was able to robustly distinguish MPNST from its PN precursor and should be tested in the setting of other hereditary cancer predisposition syndromes in the future. We nonetheless plan to enhance the technology we present here in order to boost sensitivity, for example, through deep learning following future generation of much larger cfDNA datasets, and by pairing our fragmentation- and WGS-based method with targeted deep sequencing and methylation analysis.

We also show that cfDNA tumor fraction dynamics appear to anticipate and track with disease burden in MPNST patients. CNAs in plasma cfDNA have been previously shown to correlate with radiographic burden of disease in established cancer patients [58]. Our study, similarly, showed significant correlation between plasma tumor fraction and radiographic tumor burden (**Fig 5A**). Additionally, for individual patients with serial plasma samples and serial imaging studies, dynamic changes in cfDNA tumor fraction predicted changes in tumor burden and disease state (**Figs 5B–8, S3 Fig**). These findings highlight the potential for fragment size-selected ULP-WGS surveillance in NF1, not only to distinguish between premalignant and malignant tumors, but also to serve as a real-time biomarker to track treatment response and to improve detection of MRD following local disease control. Multi-institutional prospective validation and evaluation in cfDNA-guided interventional trials is warranted.

Limitations of our study include a modest MPNST cohort size. Indeed, landmark publications on MPNST genomics comprise of whole genome sequencing (WGS)/whole exome sequencing (WES) cohorts ranging from 7 to 15 patients [43,45], comparable to our MPNST cohort size obtained from prospectively enrolling from 2 major NF1 referral centers. Still, due to our modest study size, we were unable to power a held-out validation cohort (see **Methods**). To address this, we validated our data using a leave-one-out cross-validation framework, which we show retains an overall accuracy of 75%. Ultimately, a much larger multi-institutional collaboration will be required to validate our method and the MPNST versus PN tumor fraction cutpoint. A second important limitation of this study was inconsistent testing and treatment protocols across cohort participants. Imaging data was at clinician discretion using a variety of modalities and time points. Germline NF1 testing was not conducted in all patients, and not all patients met NIH NF1 criteria. While lack of uniform clinical care likely reduced our ability to differentiate disease states, it also reflects real-world diversity in treatment expected in any large MPNST cohort outside of a dedicated prospective trial.

In conclusion, our findings suggest that cfDNA fragment analysis followed by ULP-WGS can noninvasively detect MPNST and distinguish it from its benign precursor lesion in NF1 patients. To our knowledge, these results represent the first evidence of a liquid biopsy test to capably differentiate between malignant and premalignant tumors in a heritable cancer predisposition syndrome. Application of this liquid biopsy technology has the potential to adjudicate equivocal imaging, serve as an MRD and treatment response biomarker, and, most importantly, facilitate the early detection of MPNST. These advances are critical for improving the substantial morbidity and mortality associated with these aggressive tumors in patients with this common cancer predisposition syndrome.

## Supporting information

S1 Fig Genome-wide CNAs by specimen type. Genome-wide CNAs assessed in 4 specimen types from a single MPNST patient (sar081): tumor tissue DNA, pretreatment blood plasma cfDNA, posttreatment cfDNA, and germline DNA from pretreatment PBMCs. Log_2_ of copy number ratio is shown across the genome. Color scale depicts estimated copy number within the tumor fraction as determined by ichorCNA (**Methods**). cfDNA, cell-free DNA; CNA, copy number alteration; MPNST, malignant peripheral nerve sheath tumor; PBMC, peripheral blood mononuclear cell.
(TIF)

S2 Fig Plasma cfDNA fragment sizes in MPNST patients are shorter than in PN patients or healthy controls. (**A**) Fragment size distributions of cfDNA from healthy donors, PN, and MPNST patients (**Methods**). cfDNA fragment sizes in MPNST patients were significantly shorter than from PN patients (*D* = 0.032, *P* < 0.001) or healthy donors (*D* = 0.062, *P* < 0.001) by two-sample Kolmogorov–Smirnov testing. (**B**) Log_2_ ratio of the differences in cfDNA fragment sizes from patients with MPNST versus PN with the dashed line indicating the upper boundary used for in silico size selection (150 bp). For panel A, all plasma samples in the study were analyzed (16 healthy, 23 PN, 46 MPNST), and in panel B, all PN (*n =* 23) and MPNST (*n* = 46) plasma samples were analyzed. bp, base pairs; cfDNA, cell-free DNA; MPNST, malignant peripheral nerve sheath tumor; PN, plexiform neurofibroma.
(TIF)

S3 Fig Plasma tumor fraction changes dynamically with imaging in MPNST patients. Overlaid plots of cfDNA tumor fraction (red) and SLD (blue) for MPNST patients tracked with serial plasma analysis (see also **Fig 5B**). cfDNA, cell-free DNA; MPNST, malignant peripheral nerve sheath tumor; SLD, sum of longest tumor diameters as determined by RECIST 1.1 criteria.
(TIF)

S4 Fig Higher plasma ULP-WGS sequencing depth improves the ability to differentiate MPNST from PN. Comparison of ROC summary statistics from 5 million paired reads (approximately 0.3× coverage) versus 10 million paired reads (approximately 0.6× coverage) followed by size selection of 90–150 bp cfDNA fragments. AUC, area under the curve; bp, base pairs; cfDNA, cell-free DNA; MPNST, malignant peripheral nerve sheath tumor; PN, plexiform neurofibroma; ROC, receiver operating characteristic; ULP-WGS, ultra-low-pass whole genome sequencing.
(TIF)

S1 Table Details of all sequencing libraries in this study. Characteristics, sequencing statistics, and inferred tumor fraction for each sequenced library.
(XLSX)

S2 Table MPNST patient characteristics. Overview of characteristics for patients with malignant peripheral nerve sheath tumor (MPNST).
(XLSX)

S3 Table Plexiform neurofibroma patient characteristics. Overview of characteristics for patients with benign plexiform neurofibroma.
(XLSX)

S4 Table Healthy adult donor characteristics. Overview of healthy adult donors in this study.
(XLSX)

S5 Table Confusion matrix comparing cfDNA assay performance to pretreatment MPNST vs. PN diagnosis. Confusion matrix for pretreatment MPNST versus PN using the Youden’s index-optimized cutpoint for cfDNA-based prediction.
(XLSX)

S6 Table Comparison of diagnostic tests’ ability to distinguish MPNST from PN. Comparison of assay performance in the current study to published sensitivity and specificity for standard-of-care MPNST diagnostic tests.
(XLSX)

S7 Table Assay performance with alternative ROC cutpoints and time points. Comparison of different cutpoints and conditions for the receiver operating characteristic (ROC) curve predicting MPNST vs. plexiform neurofibroma clinical status. Pretreatment represents the baseline cfDNA data, while serial analysis represents the highest plasma-derived tumor fraction detected on serial analysis.
(XLSX)

S8 Table Multivariate logistic regression predicting MPNST vs. plexiform neurofibroma clinical status. Multivariate logistic regression for predicting diagnosis with covariates being plasma tumor fraction, age, sex, and institution.
(XLSX)

## Abbreviations

AUC: area under the curve
cfDNA: cell-free DNA
CNA: copy number alteration
ctDNA: circulating tumor DNA
FFPE: formalin-fixed paraffin-embedding
IQR: interquartile range
MPNST: malignant peripheral nerve sheath tumor
MRD: minimal residual disease
NCI: National Cancer Institute
NF1: neurofibromatosis type 1
PBMC: peripheral blood mononuclear cell
PN: plexiform neurofibroma
ROC: receiver operating characteristic
SLD: sum of longest tumor diameters
ULP-WGS: ultra-low-pass whole genome sequencing
WES: whole exome sequencing
WGS: whole genome sequencing
WUSTL: Washington University in St. Louis
