# Supplementary figures and images for: Cell-free DNA ultra-low-pass whole genome sequencing to distinguish malignant peripheral nerve sheath tumor (MPNST) from its benign precursor lesion: A cross-sectional study
Source: PLoS Med. 2021 Aug 31;18(8):e1003734. doi: 10.1371/journal.pmed.1003734 (PMC8407545; doi:10.1371/journal.pmed.1003734)

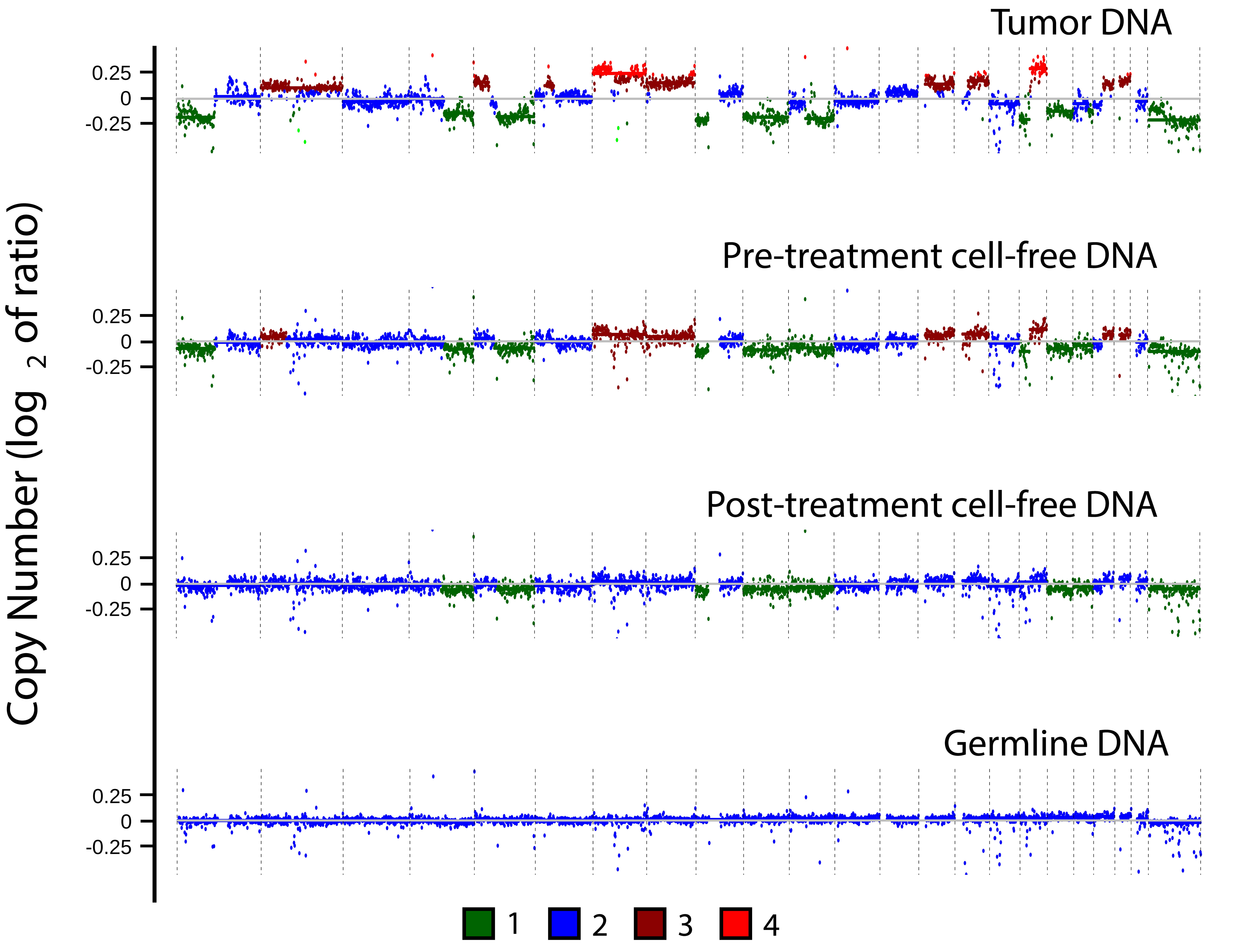

Supplement: S1 Fig — Genome-wide CNAs assessed in 4 specimen types from a single MPNST patient (sar081): tumor tissue DNA, pretreatment blood plasma cfDNA, posttreatment cfDNA, and germline DNA from pretreatment PBMCs. Log2 of copy number ratio is shown across the genome. Color scale depicts estimated copy number within the tumor fraction as determined by ichorCNA (Methods). cfDNA, cell-free DNA; CNA, copy number alteration; MPNST, malignant peripheral nerve sheath tumor; PBMC, peripheral blood mononuclear cell. (TIF) [file pmed.1003734.s001.tif]

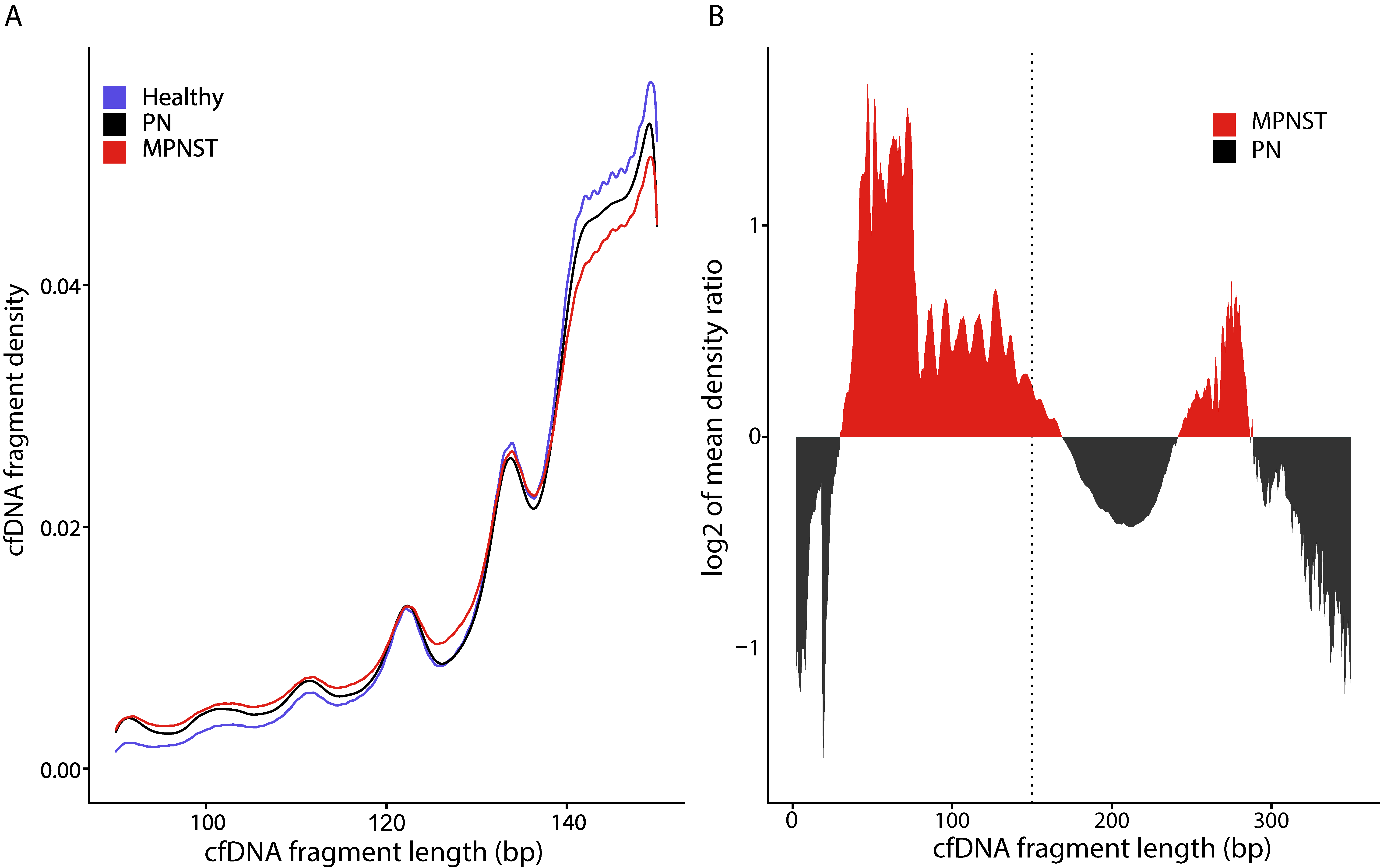

Supplement: S2 Fig — (A) Fragment size distributions of cfDNA from healthy donors, PN, and MPNST patients (Methods). cfDNA fragment sizes in MPNST patients were significantly shorter than from PN patients (D = 0.032, P < 0.001) or healthy donors (D = 0.062, P < 0.001) by two-sample Kolmogorov–Smirnov testing. (B) Log2 ratio of the differences in cfDNA fragment sizes from patients with MPNST versus PN with the dashed line indicating the upper boundary used for in silico size selection (150 bp). For panel A, all plasma samples in the study were analyzed (16 healthy, 23 PN, 46 MPNST), and in panel B, all PN (n = 23) and MPNST (n = 46) plasma samples were analyzed. bp, base pairs; cfDNA, cell-free DNA; MPNST, malignant peripheral nerve sheath tumor; PN, plexiform neurofibroma. (TIF) [file pmed.1003734.s002.tif]

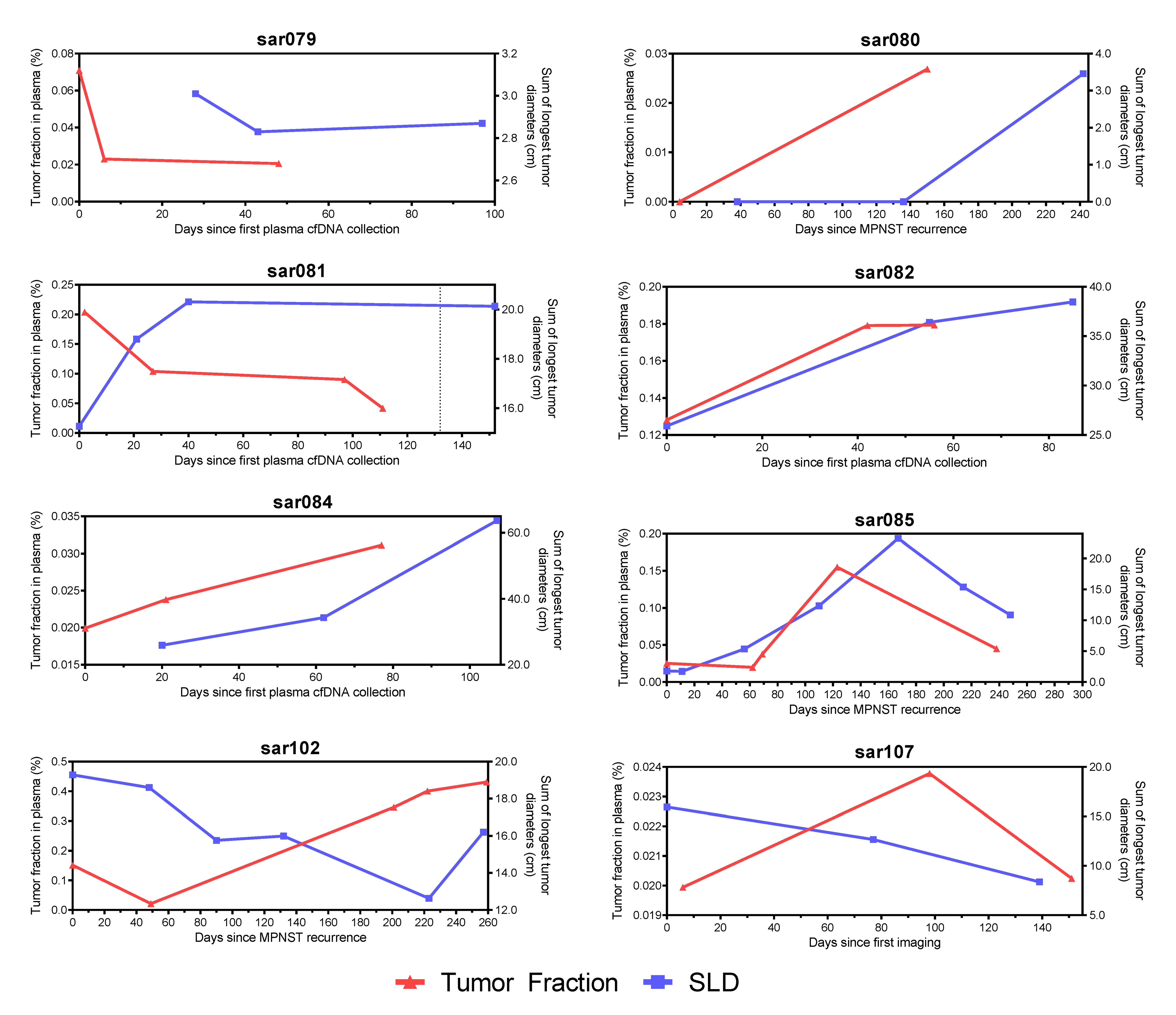

Supplement: S3 Fig — Overlaid plots of cfDNA tumor fraction (red) and SLD (blue) for MPNST patients tracked with serial plasma analysis (see also Fig 5B). cfDNA, cell-free DNA; MPNST, malignant peripheral nerve sheath tumor; SLD, sum of longest tumor diameters as determined by RECIST 1.1 criteria. (TIF) [file pmed.1003734.s003.tif]

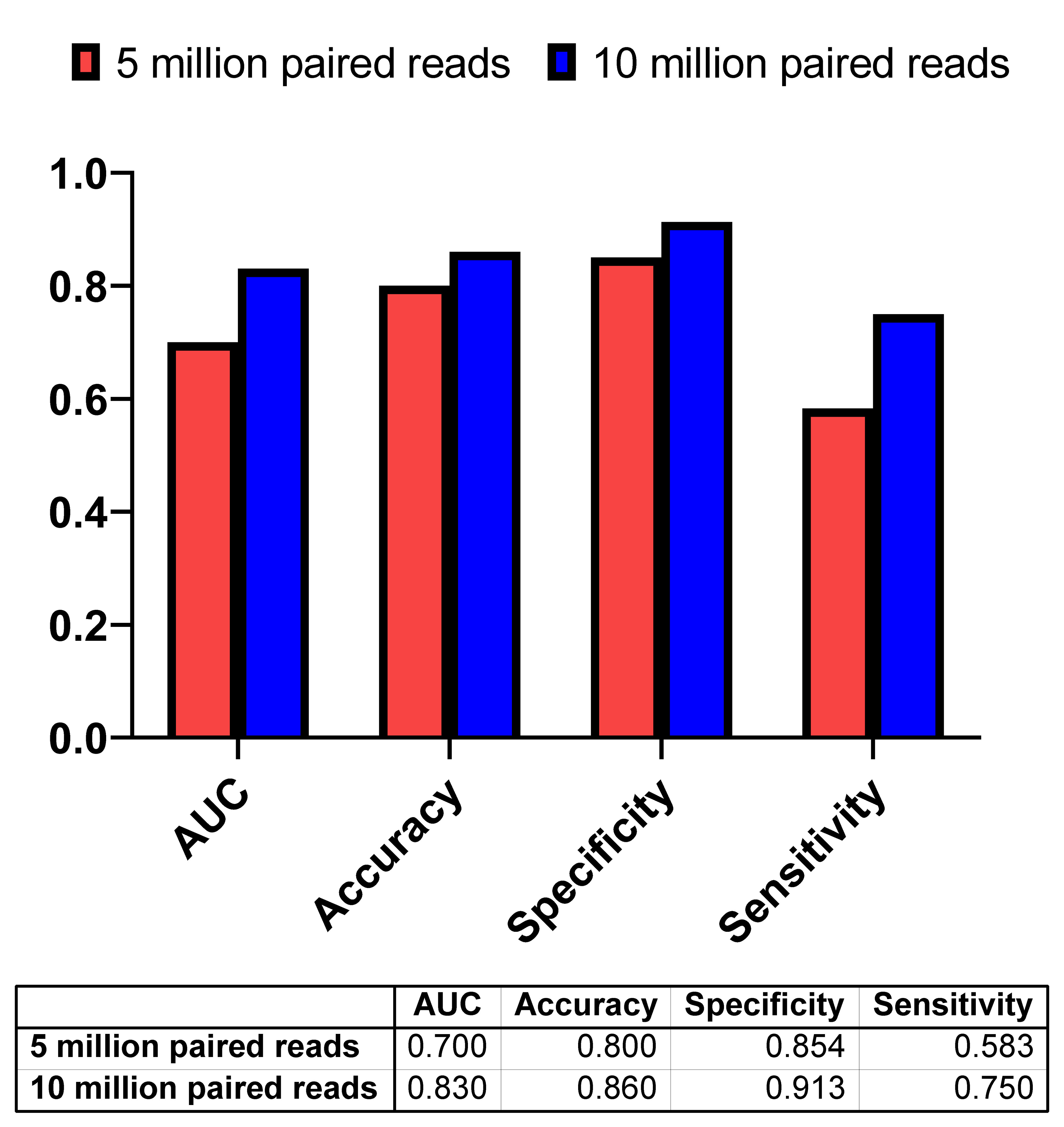

Supplement: S4 Fig — Comparison of ROC summary statistics from 5 million paired reads (approximately 0.3× coverage) versus 10 million paired reads (approximately 0.6× coverage) followed by size selection of 90–150 bp cfDNA fragments. AUC, area under the curve; bp, base pairs; cfDNA, cell-free DNA; MPNST, malignant peripheral nerve sheath tumor; PN, plexiform neurofibroma; ROC, receiver operating characteristic; ULP-WGS, ultra-low-pass whole genome sequencing. (TIF) [file pmed.1003734.s004.tif]
